# A Glycosyl Hydrolase 30 Family Xylanase from the Rumen Metagenome and Its Effects on In Vitro Ruminal Fermentation of Wheat Straw

**DOI:** 10.3390/ani14010118

**Published:** 2023-12-28

**Authors:** Longzhang Tang, Xiaowen Lei, Kehui Ouyang, Lei Wang, Qinghua Qiu, Yanjiao Li, Yitian Zang, Chanjuan Liu, Xianghui Zhao

**Affiliations:** 1 Jiangxi Province Key Laboratory of Animal Nutrition, Engineering Research Center of Feed Development, College of Animal Science and Technology, Jiangxi Agricultural University, Nanchang 330045, China; tanglz202105@163.com (L.T.); ouyangkehui@sina.com (K.O.); rcauqqh@cau.edu.cn (Q.Q.); yanjiaoli221@163.com (Y.L.); zangyitian1@126.com (Y.Z.); chanjuanhx@163.com (C.L.); 2 Ganzhou Animal Husbandry and Fisheries Research Institute, Ganzhou 341000, China; 18907977270@163.com; 3 Shandong Institute for Food and Drug Control, Jinan 250101, China; wl3613@126.com

**Keywords:** GH30 xylanase, rumen metagenome, wheat straw, ruminal fermentation

## Abstract

**Simple Summary:**

Wheat straw is commonly utilized as a roughage source for ruminants; however, its main challenge lies in its limited rumen digestibility, leading to insufficient conversion into animal products following ingestion. Efforts must be made to explore methods for enhancing the rumen digestibility of wheat straw. The remarkable efficacy, specificity, and gentle operating conditions exhibited by biological enzymes in the processing and utilization of agricultural straws have engendered considerable interest among researchers. Hence, the identification of enzymes that can efficiently enhance the digestion of wheat straw in the rumen is of utmost significance. This study investigated a xylanase called RuXyn, which is a member of the GH 30 family, derived from the rumen metagenome. The findings demonstrate that the utilization of RuXyn can significantly enhance the ruminal digestibility of wheat straw by approximately 10 percentage points. This outcome signifies the emergence of a novel and highly efficient enzyme preparation that holds promise for the effective utilization of wheat straw, a by-product of crop production, in ruminants.

**Abstract:**

The challenge of wheat straw as a ruminant feed is its low ruminal digestibility. This study investigated the impact of a xylanase called RuXyn, derived from the rumen metagenome of beef cattle, on the in vitro ruminal fermentation of wheat straw. RuXyn encoded 505 amino acids and was categorized within subfamily 8 of the glycosyl hydrolase 30 family. RuXyn was heterologously expressed in *Escherichia coli* and displayed its highest level of activity at pH 6.0 and 40 °C. RuXyn primarily hydrolyzed xylan, while it did not show any noticeable activity towards other substrates, including carboxymethylcellulose and Avicel. At concentrations of 5 mM, Mn^2+^ and dithiothreitol significantly enhanced RuXyn’s activity by 73% and 20%, respectively. RuXyn’s activity was almost or completely inactivated in the presence of Cu^2+^, even at low concentrations. The main hydrolysis products of corncob xylan by RuXyn were xylopentose, xylotriose, and xylotetraose. RuXyn hydrolyzed wheat straw and rice straw more effectively than it did other agricultural by-products. A remarkable synergistic effect was observed between RuXyn and a cellulase cocktail on wheat straw hydrolysis. Supplementation with RuXyn increased dry matter digestibility; acetate, propionate, valerate, and total volatile fatty acid yields; NH_3_-N concentration, and total bacterial number during in vitro fermentation of wheat straw relative to the control. RuXyn’s inactivity at 60 °C and 70 °C was remedied by mutating proline 151 to phenylalanine and aspartic acid 204 to leucine, boosting activity to 20.3% and 21.8% of the maximum activity at the respective temperatures. As an exogenous enzyme preparation, RuXyn exhibits considerable potential to improve ruminal digestion and the utilization of wheat straw in ruminants. As far as we know, this is the first study on a GH30 xylanase promoting the ruminal fermentation of agricultural straws. The findings demonstrate that the utilization of RuXyn can significantly enhance the ruminal digestibility of wheat straw by approximately 10 percentage points. This outcome signifies the emergence of a novel and highly efficient enzyme preparation that holds promise for the effective utilization of wheat straw, a by-product of crop production, in ruminants.

## 1. Introduction

After the harvesting of wheat, wheat straw is a widely available agricultural by-product. It is estimated that the United States, Europe, and China produce around 30–40, 200, and 140 million tons of wheat straw annually [1,2]. Wheat straw is a fiber-rich lignocellulosic substrate, containing 35–40% cellulose, 30–35% hemicellulose, and 10–15% lignin, and is thus considered a renewable, easily available, cheap, and environment-friendly by-product [3]. Wheat straw has found extensive applications in biofuel, animal feed, and paper products due to these attributes [3,4]. As an animal feed, wheat straw is mainly used for ruminants because they can degrade and utilize fiber via their rumen microorganisms [5]. Despite this, the ruminal fiber digestibility of wheat straw is relatively low (often <50%) [6], resulting in about 20% of wheat straw being discarded or directly burned in the field every year in China [2]. The intricate interconnected structure of cellulose, hemicellulose, and lignin is a significant factor contributing to its limited digestibility. This structure hinders the access of rumen microorganisms and hydrolytic enzymes to the inner part of plant fibers, consequently decreasing the quantity of fiber accessible for microbial attachment and digestion [7]. Various physical, chemical, and biological pretreatments have been used to break the lignocellulosic barrier and encourage cellulose and hemicellulose fermentation in the rumen [8]. For example, ammonia fiber expansion, steam explosion, and alkaline treatment can destroy lignocellulosic bonds, increase effective fiber surface area, and improve the ruminal and total tract digestibility of wheat straw in ruminants [6,9,10]. However, these methods require expensive equipment, are energy-intensive, and pose high risks to animals and the environment, especially alkaline treatment [11,12]. Furthermore, pretreatment of wheat straw with white-rot fungi can improve ruminal digestibility, although there are several disadvantages, such as a long culture period, loss of cellulose and hemicellulose during cultivation, high bacterial contamination, and substrate colonization by the inoculum [12,13].

On a dry-weight basis, wheat straw contains approximately 20–26% xylan [14,15,16]. Previous studies have reported that enhanced xylan digestion or removal can disrupt the xylan–lignin matrix and improve straw utilization. Notably, the removal of xylan from wheat straw reduces the particle size, deforms the cell shape, fractures the cell wall, and increases the porosity and specific surface area of wheat straw, thereby improving cellulose digestibility [16]. Xylanase plays a pivotal role as the primary enzyme in the process of xylan degradation [17]. The addition of xylanase to lignocellulosic substrates not only degrades xylan in hemicellulose but also improves the hydrolysis efficiency of cellulose by increasing the accessibility of cellulose to cellulase [18].

The inclusion of xylanases in ruminant diets with the aim of enhancing the ruminal degradation of straw has been investigated, yet the findings have not yielded conclusive results. Several studies have shown that the addition of xylanase can improve the ruminal fermentation of wheat straw by enhancing fiber degradation and volatile fatty acid (VFA) production [19,20], while other studies reported no significant positive effects [21]. Inconsistent results have also been reported for corn straw [22]. These discrepancies may be explained by various factors, such as enzyme source, supplement dosage, and application [23].

Rumen microorganisms secrete a variety of lignocellulosic enzymes, including xylanase, to decompose lignocellulosic substrates [24]. Hence, the microorganisms in the rumen have the capacity to act as a valuable source of genes for the identification of xylanases that are highly suitable for breaking down wheat straw. Importantly, the screening of xylanases using microorganisms does not require consideration of the adaptability of enzymes to conditions in the rumen environment, such as pH, temperature, and resistance to rumen proteases. In addition, high-throughput sequencing technologies such as metagenomics allows for easier prediction, cloning, and identification of microbial and enzyme genes [25]. According to metagenomic sequencing, many novel xylanase genes have been mined and identified in the rumen [26]. However, these genes represent only a small subset of possible genes, and those that have not yet been identified may show very different biochemical characteristics according to their amino acid sequence. Hence, it is crucial to detect novel xylanases for the production of ruminants using these genetic materials. Through the examination of metagenomic data (NCBI accession number PRJNA806344) from the rumen microbiota of beef cattle, we have discovered a xylanase gene that falls under the glycosyl hydrolase 30 family (GH30). This gene is responsible for the degradation of wheat straw. The identified rumen xylanase gene (*RuXyn*) was heterologously expressed in *Escherichia coli*, and its properties and effects on the hydrolysis and in vitro ruminal fermentation of wheat straw were investigated.

## 2. Materials and Methods

### 2.1. Gene Cloning and Recombinant Plasmid Construction

Details on DNA extraction, metagenomic sequencing, and data analysis of rumen microorganisms are provided in our previous study [27]. All metagenomic data were deposited in the NCBI database (accession number PRJNA806344). The *RuXyn* gene was cloned by polymerase chain reaction (PCR) using template liquid-phase cDNA and primers *RuXyn*F (CTGGTGCCGCGCGGCAGCCATATGATGAGAAAAATTATTCTTTC) and *RuXyn*R (CAGTGGTGGTGGTGGTGGTGCTCGAGCATGATAACCTTTCTGGAAGTAC). The primers have homologous sequences to the pET-28a vector. The PCR reaction mixtures were preheated to 95 °C for 3 min, followed by 35 amplification cycles (denaturing at 95 °C for 15 s, annealing at 55 °C for 15 s, and extension at 72 °C for 25 s), with a final extension at 72 °C for 5 min. The PCR fragments were ligated into the pET-28a vector using a Hieff Clone^®^ Plus One-Step Cloning Kit (10911ES20; Yeasen Biotechnology (Shanghai) Co., Ltd., Shanghai, China). The resultant plasmid was designated as pET-*RuXyn* and transformed into competent *E. coli* DH5α cells (11802ES80; Yeasen Biotechnology (Shanghai) Co., Ltd., Shanghai, China) by heat shock. The positive clones were screened by PCR amplification, extracted, and sequenced by DNA Sequencing. The sequence data were deposited in GenBank (accession number ON400054).

### 2.2. Sequence Analysis

*RuXyn* gene and amino acid sequences were aligned online (https://www.ebi.ac.uk/services, accessed on 24 April 2022) using the ENA Sequence Database and UniProt Knowledgebase, respectively. The phylogenetic tree of RuXyn was constructed using 50 similar sequences obtained from the results, employing the MEGA X version 10.2.6 software (https://www.megasoftware.net/, accessed on 24 April 2022). Multiple sequence alignments were performed by ClustalW (https://www.genome.jp/tools-bin/clustalw, accessed on 27 April 2022). The Conserved Domain Database (CDD) from NCBI was used to predict the conserved domain of RuXyn. Homologous modeling of RuXyn was performed by Phyre2 server (http://www.sbg.bio.ic.ac.uk/phyre2/, accessed on 19 May 2022) using 5CXP as the template.

### 2.3. Expression and Purification of RuXyn

The competent *E. coli* BL21(DE3) cells were subjected to transformation with the recombinant pET-RuXyn plasmid. Subsequently, the resulting transformants were cultivated on LB agar plates supplemented with kanamycin at 37 °C. The transformants confirmed by PCR were inoculated in 2 mL of LB liquid medium. Cells that had been cultured overnight (250 μL) were transferred into 25 mL of LB medium for shaking culture at 37 °C until the optical density at 600 nm reached approximately 0.8. Expression of RuXyn was then induced by isopropyl-β-D-thiogalactoside (IPTG) to a final concentration of 0–0.8 mM at 20 °C for 20 h. Simultaneously, *E. coli* BL21(DE3) cells that were not transformed were grown alongside as a control. The cultures underwent centrifugation at a speed of 8000 rpm for 15 min. Following this, the liquid portion was discarded, whereas the solid portions were immersed in phosphate-buffered saline (PBS) and crushed using ultrasonic waves in an ice-water bath. Following centrifugation, the supernatant containing RuXyn was subjected to analysis using 12.5% sodium dodecyl-sulfate polyacrylamide gel electrophoresis (SDS-PAGE) stained with Coomassie Blue, as well as Western blotting employing antibodies specific to the His-tag (66005-1-Ig, Proteintech Group, Inc., Chicago, IL, USA). The His-tag was located at the C-terminus of the RuXyn structure. Purification of RuXyn was performed using Ni-charged affinity chromatography. In summary, the supernatant obtained through ultrasonication and centrifugation was appropriately diluted with a binding buffer (pH 8.0). This buffer consisted of 0.05 M sodium dihydrogen phosphate and 0.3 M sodium chloride. Subsequently, the diluted solution was loaded onto a 5 mL Ni-charged column, which was part of a low-pressure chromatography system (Biologic LP, Bio-Rad, Hercules, CA, USA) operating at a flow rate of 1.0 mL/min. The bound RuXyn was initially washed with binding buffer containing 0.02 M imidazole and later eluted using binding buffer containing 0.25 M imidazole. The liquid carrying RuXyn was gathered and measured through the utilization of a Bradford Protein Assay Kit (Sangon Biotech, Shanghai, China) to assess its content. Subsequently, SDS-PAGE was conducted to validate the purity of the RuXyn protein.

### 2.4. Characterization of RuXyn

The characteristics of RuXyn that vary with pH were analyzed using 0.1 M citric acid-disodium hydrogen phosphate (pH 3.0–8.0) or Tris-HCl buffer (pH 8.0–9.0) at a temperature of 40 °C for a duration of 30 min, with 1% corncob xylan serving as the substrate. The released reducing sugars were analyzed using a reagent containing alkaline 3,5-dinitrosalicylic acid. Likewise, the impact of temperature on RuXyn’s function was assessed within the range of 30–70 °C, alongside the ideal pH. The acquired activities were represented as relative activities, with a maximum activity set at 100%. To assess the stability of RuXyn, its resistance to changes in pH and temperature was evaluated. The enzyme was subjected to a preincubation step at temperatures ranging from 30 to 70 °C for 5 and 90 min, and at a pH range of 3.0 to 9.0 for 1 h at 4 °C. Subsequently, the remaining activity was measured under the ideal conditions of pH 6.0 and 40 °C.

To analyze the substrate specificity of RuXyn, reaction mixtures containing 1% substrate were examined under optimal conditions. The substrates included corncob xylan, wheat straw xylan, corn straw xylan, wheat bran xylan, chitosan, sodium carboxymethylcellulose, Avicel, and yeast β-glucan. The xylan from corncob, wheat straw, corn straw, and wheat bran was extracted based on a previous study [28].

The absolute activity of RuXyn was determined with 1% corncob xylan as a substrate under optimal conditions for 30 min [29]. The definition of one unit of enzymatic activity (U) was the quantity of enzyme that generated 1 μmol of xylose per min. The Michaelis–Menten equation was used for nonlinear regression analysis to determine the Km and Vmax values of RuXyn, using 0.2–2.5% corncob xylan as a substrate. This analysis was performed with GraphPad Prism v5.0 (GraphPad Software Inc., San Diego, CA, USA).

To examine the durability of RuXyn against different substances like metal ions, inhibitors, and detergents, the reaction mixture was modified by adding these additives for investigation purposes. The subsequent reaction was carried out for a duration of 30 min under conditions that were considered to be the most favorable. In the absence of any additional substances, the baseline for enzymatic activity was established at 100%.

### 2.5. Hydrolysis Products of Corncob Xylan

A 1 mL reaction system consisting of 1% corncob xylan and 0.156 U RuXyn was employed under optimal conditions for 30 min. A control was established using a reaction containing inactivated RuXyn. The hydrolysis products were subsequently analyzed utilizing a high-performance liquid chromatography (HPLC) system (model D-7000, Hitachi Ltd., Tokyo, Japan), following the methodology outlined in our previous study [27]. The flow rate was adjusted to 1 mL/min, with UV detection at a wavelength of 245 nm. The mobile phase consisted of a blend of buffers A and B, i.e., 0.02 M ammonium acetate combined with 17% and 75% acetonitrile, respectively. A gradient ranging from 0 to 40% buffer B was employed over a 30 min period for the separation process. Prior to analysis, the products were subjected to derivatization using 1-phenyl-3-methyl-5-pyrazolone, as described by Li et al. (2013) [30].

### 2.6. Hydrolysis of Fiber Substrates by RuXyn

Six fiber substrates, including wheat bran, wheat straw, rice straw, corn straw, corncob, and distillers dried grains with solubles (DDGSs) from corn, were used to explore the application potential of RuXyn. Hydrolysis was carried out by incubating 5% substrate and 0.156 U RuXyn in a 1 mL reaction system under optimal conditions for 30 min, with the released reducing sugars then analyzed. A control containing the inactive enzyme was incubated simultaneously.

### 2.7. Synergistic Effects of RuXyn and Cellulase on Hydrolysis of Wheat Straw

Based on previous results, wheat straw was selected as the substrate for the following analyses: In order to examine the combined impacts of RuXyn and cellulase on the breakdown of wheat straw, a mixture containing 0.78 U of RuXyn and 5% wheat straw was subjected to preincubation in a 0.1 M citric acid buffer (5 mL, pH 6.0) at 40 °C for 90 min. Subsequently, the liquid component was discarded, and the remaining solid residues were subjected to two washes with distilled water. These residues were then utilized as the substrate for cellulase. In a parallel manner, a control group containing inactive RuXyn was subjected to a comparable incubation process.

The leftover substances were placed in a 0.1 M citric acid solution (5.0 mL, pH 5.0) with the addition of 1 mg of cellulase mixture derived from *Trichoderma* sp. (C8272, Beijing Solarbio Science & Technology Co., Ltd., Beijing, China; 10 U/mg). Afterwards, the mixture was placed in an incubator at a temperature of 37 °C for a duration of 90 min. Glucose quantification was carried out by utilizing a glucose oxidase-based commercial glucose analysis kit (A154-1-1, Nanjing Jiancheng Bioengineering Institute, Nanjing, China).

### 2.8. Hydrolysis of Wheat Straw by RuXyn

To obtain a reference dosage of RuXyn for subsequent in vitro ruminal fermentation of wheat straw, we performed wheat straw hydrolysis under different concentrations of RuXyn. A reaction mixture consisting of 5% wheat straw and RuXyn (1.56–7.80 U/g substrate) was incubated in 1.5 mL of citric acid buffer under optimal conditions for 120 min, with the released reducing sugars then determined.

### 2.9. In Vitro Ruminal Fermentation

To investigate the effects of RuXyn on the ruminal degradation of wheat straw, we performed an in vitro experiment, as per our previous study [20]. This study was approved by the Animal Care and Use Committee of Jiangxi Agricultural University (JXAULL-2023-10-19). In short, rumen liquid was collected from ruminally fistulated beef cattle fed a diet consisting of 700 g/kg rice straw and 300 g/kg concentrates before morning feeding, then filtered and mixed (1:2 *v*/*v*) with anaerobic buffer. For fermentation, 500 mg of wheat straw, 3.9 U of RuXyn/inactive RuXyn (control), and 60 mL of buffered rumen fluid were added to a 120 mL serum bottle, which was sealed and anaerobically shaken at 39 °C for 48 h. A blank containing only rumen fluid was performed under the same procedures for correcting dry matter residue in the samples. Fermentation was terminated after 48 h, and samples were collected for analysis of pH, in vitro dry matter digestibility (IVDMD), volatile fatty acids, NH_3_-N, and total bacteria following previous research [20]. Methane production was estimated based on previously reported methods [31].

### 2.10. Site-Directed Mutagenesis of RuXyn

To enhance the thermal stability of RuXyn, the Calculate Mutation Energy program (Stability) from Discovery Studio (DS) 2016 was employed to assess the mutation energy following single amino acid and double amino acid saturation mutations according to the homologous model constructed. Based on the findings, a single amino acid mutation involved the substitution of proline, the 151st amino acid, with phenylalanine in RuXyn (RuXyn^P151F^, SM-RuXyn). Furthermore, a double amino acid mutation occurred, wherein the 204th amino acid (aspartic acid) was replaced with leucine (RuXyn^P151F,N204L^, DM-RuXyn). A fast mutagenesis system (TransGen Biotech, Beijing, China) was used to carry out the mutation process according to the operation instructions with the pET-RuXyn vector as a template. The expression plasmids for SM-RuXyn and DM-RuXyn were transformed into competent *E. coli* BL21(DE3) cells. The analysis of the pH dependency, temperature dependency, and thermostability of SM-RuXyn and DM-RuXyn was conducted in accordance with the previously described methodology (Section 2.4).

### 2.11. Statistical Analyses

The statistical analysis was conducted using IBM SPSS v20 (IBM, Chicago, IL, USA). The data were analyzed by one-way ANOVA using a model as follows: Yij = µ + Ti + Ei, where Yi is the dependent variable, µ is the overall mean, Ti is the Treatment effect, and Ei is the error term. The LSD test was utilized to conduct multiple comparisons of means across treatments. A level of significance of *p* ≤ 0.05 was utilized.

## 3. Results

### 3.1. Sequence Analysis and Production of RuXyn

**Figure 1:**
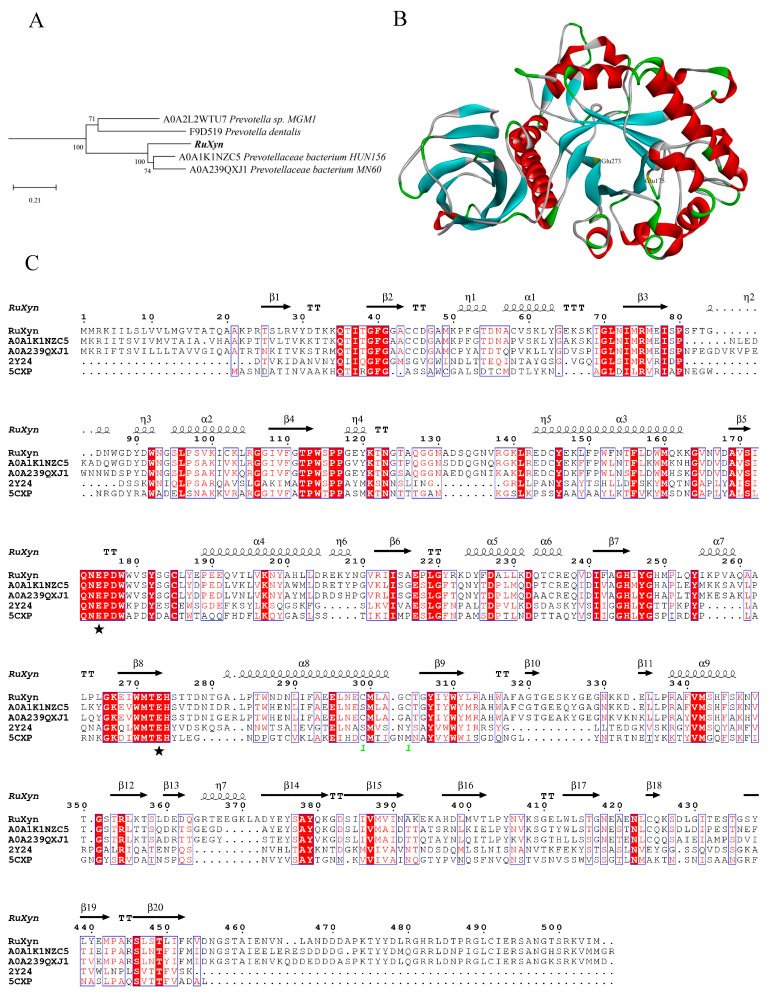
Phylogenetic tree (**A**), homology modeling (**B**), and multi-alignment analysis (**C**) of RuXyn. Putative catalytic residues (pentacle).

**Figure 2:**
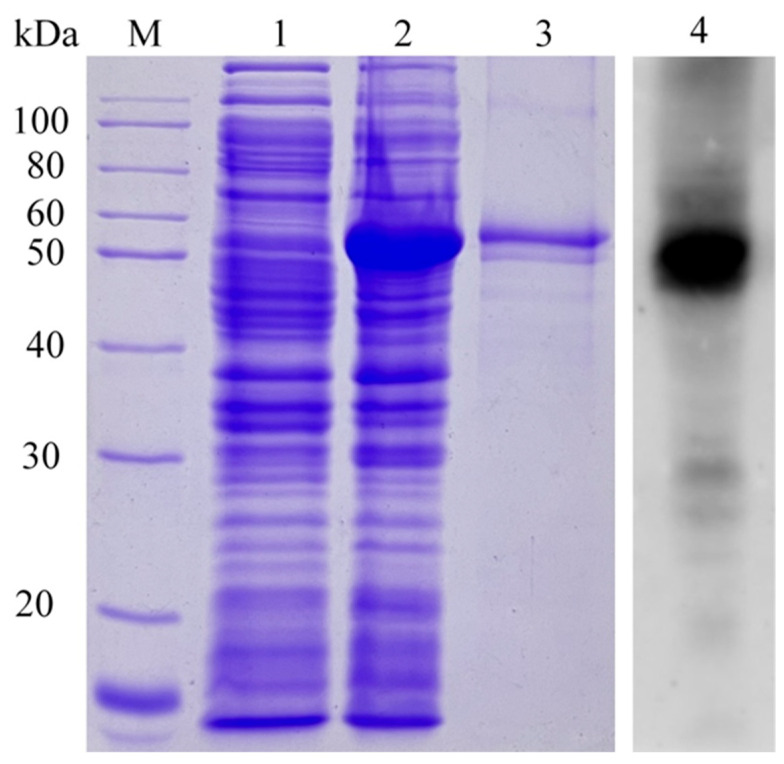
Analysis of RuXyn by SDS-PAGE and Western blotting. M, protein marker; 1, nontransformed *E. coli* BL21(DE3); 2, RuXyn transformants induced with 0.2 mM IPTG; 3, purified RuXyn; 4, Western blot analysis of supernatant from the ultrasonication of RuXyn transformants.

The *RuXyn* gene was cloned from the cDNA of microorganisms present in the rumen liquid phase, based on the coding sequence (CDS) identified through the analysis of rumen metagenomic data. Subsequent sequencing revealed that the *RuXyn* gene spans a length of 1518 base pairs and encodes a protein consisting of 505 amino acids. The predicted theoretical molecular weight of this protein is 56 kDa. A BLAST (nucleotide) analysis of EMBL-EBI (https://www.ebi.ac.uk/services, accessed on 24 April 2022) using the ENA Sequence Database with default parameters did not reveal significantly similar sequences. According to the UniProt Knowledgebase, the BLAST (protein) findings revealed that RuXyn had the highest similarity (70.8%) with a glucuronoarabinoxylan endo-1,4-beta-xylanase sequence from *Prevotellaceae bacterium* HUN156 (UniProtKB accession A0A1K1NZC5) in the GH30 family at the amino acid level. This information is shown in Supplementary Figure S1. Furthermore, RuXyn exhibited a significant resemblance to endo-1,4-beta-xylanase sequences derived from *Prevotellaceae bacterium* MN60 (65.2%), *Prevotella* sp. MGM1 (48.8%), and *Prevotella dentalis* (40.5%), which also belong to the GH30 family. Several protein sequences with low similarity (27.4–34.4%) to RuXyn were also found in the search results. Of note, many of the protein sequences mentioned above were predicted from the metagenomic data but were not categorized by biochemical characteristics. A RuXyn phylogenetic tree was constructed using 50 protein sequences, based on the findings from the BLAST analysis. The findings indicated that RuXyn had a comparatively strong genetic connection to the glucuronoarabinoxylan endo-1,4-beta-xylanases found in *Prevotellaceae bacterium* HUN156 (UniProtKB accession A0A1K1NZC5) and *Prevotellaceae bacterium* MN60 (UniProtKB accession A0A239QXJ1) (as shown in Figure 1A and Figure S2). A CDD analysis revealed the existence of the GH30 superfamily domain ranging from 1 to 451 amino acid residues (Supplementary Figure S3). Using Phyre2 with 5CXP as the template (Figure 1B), the homology modeling technique was employed to construct the 3D configuration of RuXyn. The results showed that the predicted RuXyn structure was composed of a nine-strand aligned β-sheet structure (β_9_-domain) and a (β/α)_8_ barrel structure, with only one β-strand of the β_9_-domain formed before the polypeptide chain entered the (β/α)_8_ motif. RuXyn was aligned with several proteins that had comparable amino acid sequences or structures. In the amino acid sequence of RuXyn (Figure 1C), there were several residues (173–178, 270–274) that were strictly conserved. RuXyn was heterologously expressed in *E. coli* BL21(DE3), with IPTG used to induce RuXyn expression. A distinct protein band with an approximate 54 kDa was identified through SDS-PAGE, and subsequent Western blot analysis confirmed its identity as the RuXyn protein (Figure 2). The observed molecular weight was consistent with the theoretical molecular weight of RuXyn, including the tagged protein. The concentration of purified RuXyn obtained was 99.5 μg/mL.

### 3.2. Characterization of RuXyn

**Figure 3:**
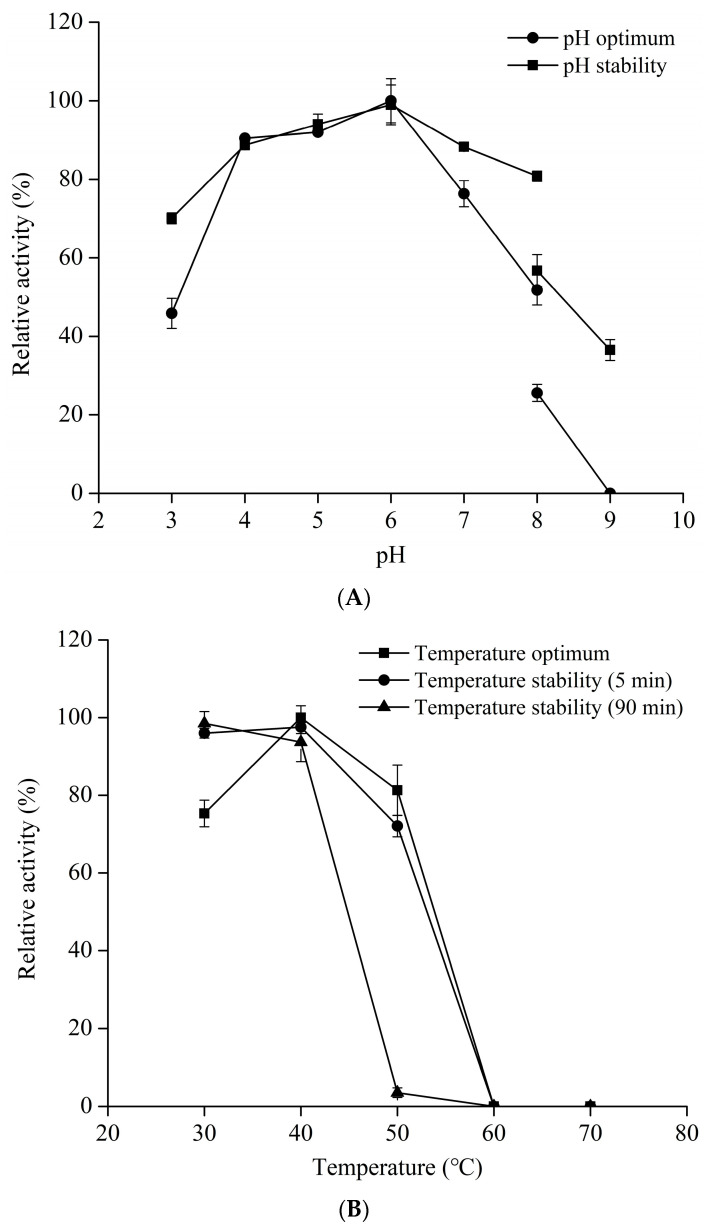
pH dependence (**A**), temperature dependence (**B**), pH stability (**A**), and thermostability (**B**) of RuXyn. Each value represents the mean ± standard deviation of three replicates.

RuXyn displayed enzymatic function across a wide pH spectrum (4.0–8.0), reaching its peak at pH 6.0 (Figure 3A). RuXyn exhibited a high level of activity, surpassing 76%, within a pH range of 4 to 7.0. The pH stability results showed that RuXyn can still retain more than 70% of its maximum activity after pretreatment at pH 3.0–8.0 for 1 h. The temperature dependence of RuXyn at pH 6.0 is shown in Figure 3B. Purified RuXyn was most active at 40 °C and retained more than 60% of its maximum activity at 30 °C and 50 °C. Nevertheless, temperatures exceeding 60 °C resulted in the complete inactivation of the enzyme. For thermostability, RuXyn was preincubated at 30–70 °C for 5 min and 90 min, and residual activity was measured under optimal conditions. The enzyme retained over 70% of its initial activity at 30–50 °C for 5 min, but residual activity could not be detected when pretreatment temperatures exceeded 60 °C. When RuXyn underwent preincubation at 50 °C for 90 min, its remaining activity was reduced to less than 5% of its peak level. These results indicate that RuXyn does not exhibit excellent high-temperature resistance.

RuXyn showed a strong preference for xylan substrates including corncob xylan (specific activity: 5.2 U/mg), wheat straw xylan (specific activity: 5.0 U/mg), corn straw xylan (specific activity: 8.2 U/mg), and wheat bran xylan (specific activity: 9.6 U/mg), but no activity for the other substrates. The kinetic parameters of RuXyn, including Km, Vmax, Kcat, and Kcat/Km, were determined using corncob xylan as a substrate. The values obtained were 28.78 g·L^−1^, 35.87 μmol xylose·min^−1^·mg^−1^ protein, 32.28/s, and 1.12 L·s^−1^·g^−1^, respectively.

Table 1 presents the observed impacts of metal ions, inhibitors, and detergents on the activity of RuXyn. RuXyn showed variable responses to the metal irons. Significantly, Zn^2+^, Mg^2+^, and Cu^2+^ effectively suppressed the activity of RuXyn, even at concentrations as low as 1 mM, with Cu^2+^ completely deactivating the enzyme. In contrast, Mn^2+^ at 1 and 5 mM markedly increased RuXyn’s activity by 59% and 73%, respectively, while Ca^2+^ slightly increased RuXyn’s activity. RuXyn experienced different levels of inhibition from Tween-20, Triton X-100, β-mercaptoethanol, sodium dodecyl-sulfate (SDS), and ethylenediaminetetraacetic acid (EDTA). The enzyme activity was reduced by 26% due to Tween-20, Triton X-100, and β-mercaptoethanol, whereas SDS and EDTA resulted in a 57% reduction in enzyme activity. RuXyn’s activity was not affected by dithiothreitol at 1 mM but was increased by 20% in the presence of dithiothreitol at 5 mM.

### 3.3. Hydrolysis Products of Corncob Xylan by RuXyn

**Figure 4:**
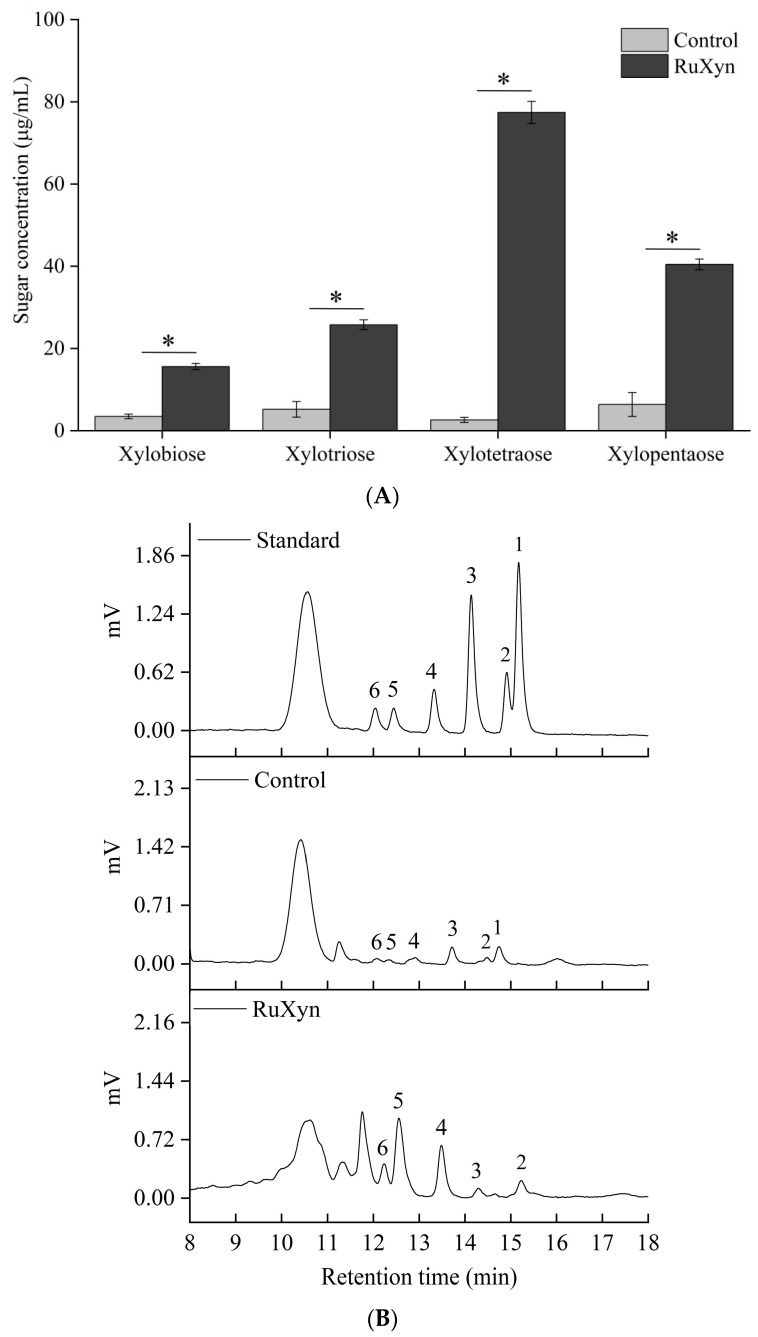
Product analysis of corncob xylan by RuXyn. (**A**) Each value represents the mean and standard deviation of three replicates. (**B**) HPLC peaks: 1—xylose, 2—xylobiose, 3—glucose, 4—xylotriose, 5—xylotetraose, and 6—xylopentaose. Significant differences are denoted by asterisks (*p* ≤ 0.05).

To investigate the types of xylooligosaccharides (XOSs) produced by RuXyn, we analyzed the hydrolysis products of corncob xylan. Relative to the control, RuXyn treatment increased the yields of xylobiose, xylotriose, xylotetrose, and xylopentaose by 12.1, 20.6, 74.8, and 34.1 µg/mL, respectively (Figure 4A), suggesting that xylotetrose is the main product produced, followed by xylopentaose and xylotriose. In addition, xylose was not clearly detected in the samples treated by RuXyn (Figure 4B), indicating that RuXyn can efficiently produce XOSs from corncob xylan.

### 3.4. Hydrolysis of Fiber Substrates by RuXyn

**Figure 5:**
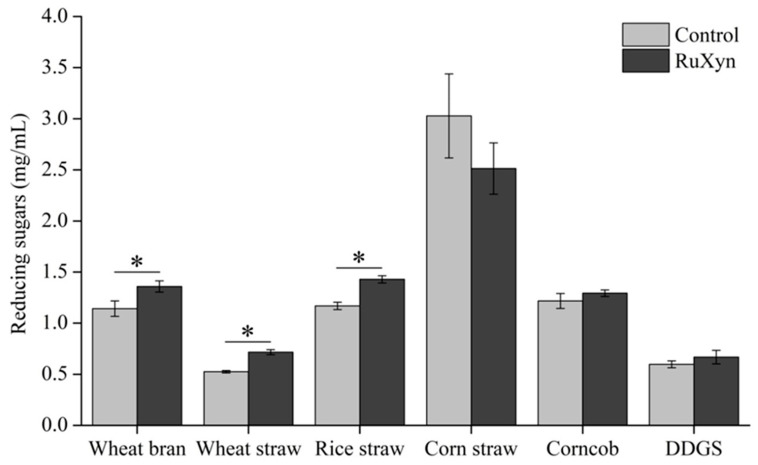
Hydrolysis of fiber substrates by RuXyn. Each value represents the mean and standard deviation of three replicates. Significant differences are denoted by asterisks (*p* ≤ 0.05).

The ability of RuXyn to hydrolyze different agricultural by-products was analyzed. The findings indicated that RuXyn greatly enhanced the breakdown of these substances, with the exception of corn straw (Figure 5). Compared to the control, RuXyn promoted the release of sugars from wheat bran, wheat straw, and rice straw by 217, 191, and 260 µg/mL, respectively. However, the hydrolysis of corn straw, corncob, and DDGS was not significantly different between RuXyn and the control (*p* > 0.05).

### 3.5. Synergistic Effects of RuXyn and Cellulase on Wheat Straw

**Figure 6:**
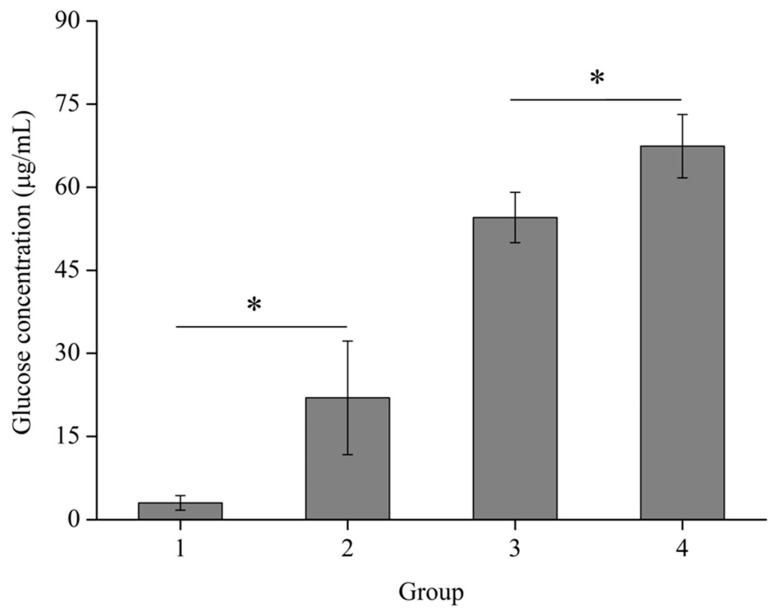
Enhanced wheat straw hydrolysis through the combined action of RuXyn and cellulase. In group 1, wheat straw was pretreated with inactive RuXyn at pH 6.0 and 40 °C for 90 min (first step), and the solid residues obtained were incubated without a cellulase cocktail at pH 5.0 and 37 °C for 90 min (second step). Compared with group 1, RuXyn was used in the first step in group 2, a cellulase cocktail was added in the second step in group 3, and RuXyn and a cellulase cocktail were sequentially used in the first and second steps, respectively, in group 4. Released glucose was determined after the second step in the four groups. Each value represents the mean and standard deviation of three replicates. Significant differences (*p* ≤ 0.05) are denoted by asterisks.

To investigate the effects of xylan removal on wheat straw cellulose, the synergistic effects of RuXyn and cellulase were analyzed. Compared to wheat straw pretreated with buffer (group 3), wheat straw pretreated with RuXyn (group 4) showed better accessibility to cellulase, enhancing glucose yield by 12.9 µg/mL during cellulase hydrolysis (*p* ≤ 0.05, Figure 6). In addition, comparing groups 1 and 2, RuXyn pretreatment increased the subsequent release of glucose from residual wheat straw (*p* ≤ 0.05).

### 3.6. Hydrolysis of Wheat Straw by RuXyn

**Figure 7:**
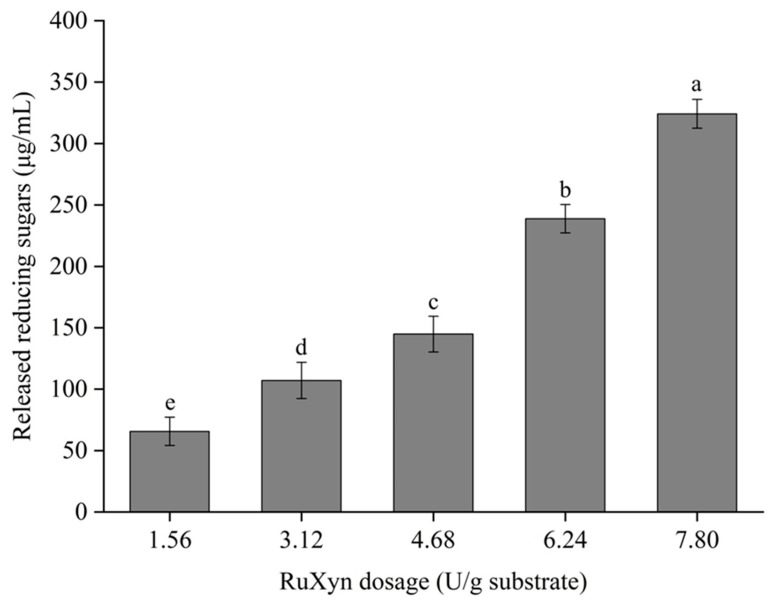
Hydrolysis of wheat straw by RuXyn. Each value represents the mean and standard deviation of three replicates. Significant differences are indicated by different lowercase letters (*p* ≤ 0.05).

The effects of different dosages of RuXyn on wheat straw hydrolysis were further investigated. The results showed that wheat straw hydrolysis was significantly enhanced with the increase in xylanase dosage (*p* ≤ 0.05, Figure 7). The released reducing sugars increased from 65 µg/mL in the 1.56 U/g substrate group to 324 µg/mL in the 7.80 U/g substrate group, indicating that the amount of substrate was sufficient for RuXyn at the current dosage range.

### 3.7. Effects of RuXyn on In Vitro Ruminal Fermentation of Wheat Straw

To investigate the potential of RuXyn to improve straw utilization in ruminants, we determined the effect of RuXyn addition to wheat straw on ruminal fermentation in vitro. Interestingly, compared to the control, IVDMD increased by 8.8% after 48 h of fermentation when supplemented with RuXyn (*p* = 0.001, Table 2). The addition of RuXyn reduced the pH (*p* = 0.007) of the fermentation liquid and increased the yields of acetate (*p* = 0.011), propionate (*p* < 0.001), valerate (*p* = 0.029), and total VFA (*p* = 0.017) by 2.4, 3.4, 3.9, and 10.4 mM, respectively. Both NH_3_-N concentration (*p* < 0.001) and total bacterial number (*p* = 0.016) also increased significantly under RuXyn supplementation.

### 3.8. Site-Directed Mutagenesis of RuXyn

**Figure 8:**
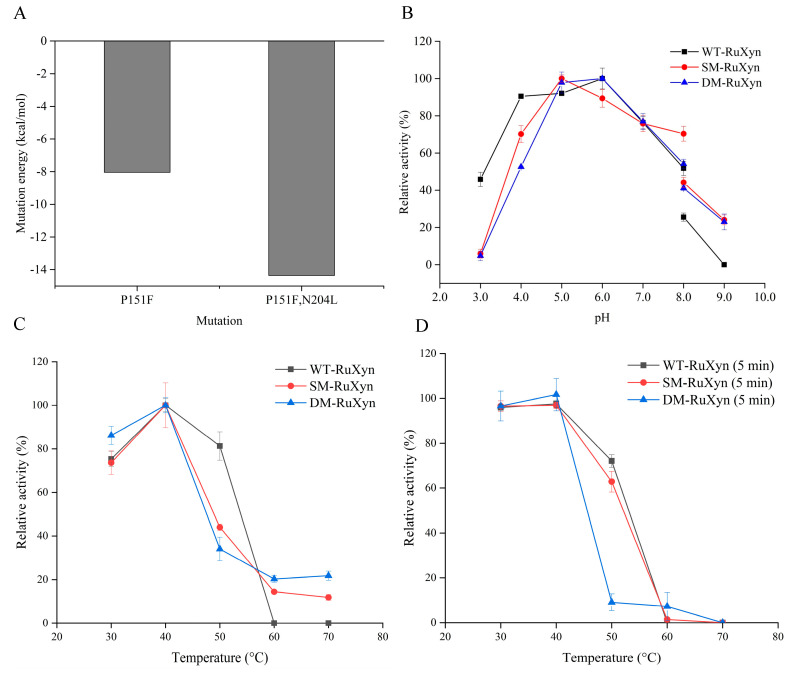
Site-directed mutagenesis of RuXyn. (**A**) Predicted mutation energy after mutating proline 151 and aspartic acid 204 to phenylalanine and leucine, respectively; (**B**) pH dependence of mutants SM-RuXyn and DM-RuXyn; (**C**) temperature dependence of mutants SM-RuXyn and DM-RuXyn; (**D**) thermal stability of mutants SM-RuXyn and DM-RuXyn.

This experiment aimed to enhance the thermal stability of RuXyn through site-directed mutation. Prior to mutation, saturation mutation of a single amino acid was conducted on RuXyn using the Calculate Mutation Energy Program (stability) of DS, and the alteration in mutation energy was observed. The findings revealed that the substitution of proline 151 with phenylalanine (RuXyn^P151F^, SM-RuXyn) resulted in a predicted mutation energy of −8.04 kcal/mol (Figure 8A), suggesting that this mutation had the potential to augment the thermal stability of RuXyn. Based on SM-RuXyn, we proceeded to perform comprehensive single amino acid mutations on various amino acids. The findings revealed that the substitution of aspartic acid 204 with leucine (RuXyn^P151F,N204L^, DM-RuXyn) resulted in a decrease in mutation energy from −8.04 to −14.36, suggesting that DM-RuXyn may have exhibited superior thermal stability compared to SM-RuXyn. In order to validate the aforementioned predictive outcomes, an analysis was conducted to examine the impacts of pH and temperature on mutants SM-RuXyn and DM-RuXyn. The findings indicated that the mutants exhibited reduced relative activity within the pH range of 3.0–4.0 while displaying enhanced relative activity within the pH range of 8.0–9.0 when compared to the original RuXyn (wild-type RuXyn, WT-RuXyn) (Figure 8B). The temperature-dependent findings indicated that, in comparison to WT-RuXyn, mutants SM-RuXyn and DM-RuXyn displayed a decrease in relative activity at 50 °C (Figure 8C). However, unlike WT-RuXyn, which exhibited no activity at 60–70 °C, the mutants demonstrated a relative activity range of 11.8–21.8%. The thermal stability analysis revealed that mutants SM-RuXyn and DM-RuXyn exhibited a decrease in residual activity after treatment at 50 °C (Figure 8D). Nevertheless, in contrast to WT-RuXyn, which displayed no discernible activity after treatment at 60 °C, the mutants still retained minimal residual activity (<10%).

## 4. Discussion

The BLAST (protein) and phylogenetic results both showed a close relationship between RuXyn and a *Prevotellaceae bacterium HUN156* xylanase belonging to the GH30 family. *Prevotella*, a dominant genus in the rumen, has the ability to break down different polysaccharides, such as xylan, through hydrolysis [32]. These results are consistent with the fact that RuXyn was derived from beef cattle rumen. A CDD analysis also detected a GH30 superfamily domain, while homology modeling predicted that the RuXyn structure contained a β_9_-domain and (β/α)_8_ barrel structure, which are typical features of GH30 [33,34], further confirming that RuXyn belongs to the GH30 family. Moreover, since the (β/α)_8_ motif in the anticipated arrangement was preceded by only a single β-strand in the β_9_-domain, it can be inferred that RuXyn, being a prokaryotic xylanase, is most probably a member of subfamily 8 within GH30 [33]. Two typical catalytic residues, E175 and E273 (marked with a pentacle in Figure 2B), were observed in the strictly conserved motifs of RuXyn [35]. Conserved residues 173–178 and 270–274 were located on a β-sheet and a turn opposite to it within the barrel structure, respectively.

RuXyn exhibited peak activity at pH 6.0 and 40 °C, resembling the parameters found in the rumen environment (pH 6.0–7.0 and temperature 39–41 °C) [36,37]. The findings align with a prior investigation where xylanases derived from a metagenomic library in the rumen displayed peak performance at a pH of 6.5 and a temperature of 40 °C but exhibited limited resistance to high temperatures [38].

Several metal ions, including Zn^2+^, Mg^2+^, and Cu^2+^, significantly inhibited RuXyn’s activity, possibly by hindering enzymatic reactions after combining with the active group (thereby causing oxidative stress and structural protein damage) or by reacting with the enzyme–substrate complex [39,40]. Mn^2+^ serves as an activator for RuXyn and exhibits a significant enhancement in RuXyn’s activity. This observation implies that Mn^2+^ potentially plays a crucial role in enzymatic activity by interacting with essential amino acid residues within the enzyme’s active site, acting as a cofactor, or inducing alterations in the tertiary structure [41,42]. These results are similar to previous reports, in which Zn^2+^, Mg^2+^, and Cu^2+^ repressed while Mn^2+^ stimulated the activities of xylanases cloned from rumen microorganisms [40,43]. Both SDS and EDTA showed significant inhibition of RuXyn’s activity. The potential inhibitory impact of SDS can be attributed to its anionic surfactant characteristics, which may induce a conformational alteration in the enzyme, resulting in its inactivation and denaturation [44,45]. The inhibitory effect of EDTA, a metal chelator, on RuXyn suggests that xylanases may require metal ion cofactors [45]. This could be confirmed by the increase in RuXyn’s activity in the presence of Mn^2+^. The stimulatory effect of dithiothreitol may be due to the prevention of sulfhydryl group oxidation, presumably from cysteine residues, thereby stabilizing the enzyme [45,46].

Our results indicated that the ability of RuXyn to degrade wheat and rice by-products was greater than that of corn-derived by-products, which may be related to the different fibrous structures of the substrates. Fiber substrates can be hydrolyzed by enzymes secreted by rumen microorganisms, which is the fundamental reason why ruminants can effectively utilize lignocellulose [24]. Similarly, other xylanases from rumen microbial metagenomes have been shown to hydrolyze several substrates used in this study [38,47]. Surprisingly, RuXyn inhibited the release of reducing sugars from corn straw rather than increasing it. However, the reason for this remains unclear.

Previous studies have shown that the removal of xylan from lignocellulosic substrates by xylanases can enhance subsequent cellulase hydrolysis [18]. This synergy between xylanase and cellulase appears to be important in ruminants due to the abundance of microbially secreted carbohydrate hydrolases, including cellulases, in the rumen [24]. In the present study, the results showed that the pretreatment of wheat straw with RuXyn enhanced subsequent wheat straw hydrolysis by cellulase, thereby exhibiting clear synergy between RuXyn and cellulase, consistent with prior research showing obvious synergistic effects between xylanase and commercial cellulase on wheat straw hydrolysis [48]. Furthermore, RuXyn pretreatment increased the subsequent release of glucose from residual wheat straw, possibly due to the disruption of cell wall components, which facilitated the leaching of soluble sugars [49].

Various studies have demonstrated that the introduction of exogenous xylanases can effectively augment the in vitro ruminal fermentation of wheat straw, leading to heightened VFA production and improved fiber digestibility [19,20], in partial agreement with our results. Here, the increased IVDMD may be related to the accelerated enzymatic hydrolysis of nutrients in wheat straw by RuXyn. These nutrients, including cellulose, hemicellulose, soluble sugars, and protein, may be more easily degraded by rumen microorganisms with the help of RuXyn, which was partially confirmed by the synergy results. In addition, RuXyn supplementation significantly increased the total VFA concentration, NH_3_-N concentration, and the 16S rDNA copy number of total bacteria. As end-products, rumen VFAs are mainly formed from the microbial fermentation of dietary carbohydrates, i.e., cellulose, hemicellulose, pectin, starch, and soluble sugars [50]. The increase in NH_3_-N concentration, which is a balance between dietary protein degradation and nitrogen uptake by rumen microorganisms, may be the result of higher dietary nitrogen degradation caused by RuXyn [20]. This was confirmed by the increased valerate concentration in the RuXyn group, as isovalerate and valerate are end-products of rumen protein degradation [51]. Exogenous fibrolytic enzymes can increase the rumen bacterial colonization of lignocellulosic substrates and accelerate substrate digestion, which may have led to the increased 16S rDNA copy number of total bacteria after RuXyn supplementation [52]. Overall, RuXyn effectively improved the ruminal fermentation of wheat straw. To the best of our knowledge, this is the first report showing that a GH30 xylanase can improve the ruminal fermentation of agricultural straw.

High-temperature resistance is generally recognized as an advantageous characteristic of enzymes. This study endeavored to modify RuXyn’s tolerance to high temperatures through the implementation of site-directed mutations. The findings indicated that, in contrast to WT-RuXyn, both the SM-RuXyn and DM-RuXyn mutants demonstrated activity when subjected to reaction conditions of 60 °C and 70 °C. Additionally, the tolerance of DM-RuXyn to 60 °C was enhanced. This observation suggested that the two aforementioned mutants exhibited a modestly beneficial impact in mitigating the inactivation of RuXyn at temperatures of 60 °C and 70 °C. However, it was important to acknowledge that this positive effect was relatively limited. Consequently, the substitution of proline 151 and aspartic acid 204 residues in RuXyn with phenylalanine and leucine, respectively, may not be the optimal approach. Additional investigation is warranted to enhance the thermal stability of RuXyn.

RuXyn did not hydrolyze cellulosic substrates, indicating its potential use in the paper and pulp industry [53]. RuXyn successfully generated XOSs from corncob xylan, which has attracted attention because of the prebiotic potential and nutritional advantages of XOSs in different animal species [54].

In summary, based on rumen metagenomics, we identified a xylanase in beef cattle expressed in *E. coli*. RuXyn exhibited maximum activity at pH 6.0 and 40 °C but showed poor thermostability. The presence of Mn^2+^ greatly enhanced RuXyn’s activity. The main hydrolysis products of corncob xylan by RuXyn were xylopentose, xylotriose, and xylotetraose. RuXyn effectively hydrolyzed wheat straw and rice straw and showed a synergistic effect with cellulase in wheat straw hydrolysis. Supplementation with RuXyn increased dry matter digestibility, the VFA yield, NH_3_-N concentration, and the total bacterial number during in vitro fermentation of wheat straw. RuXyn’s inactivity at 60 °C and 70 °C was remedied by mutating proline 151 to phenylalanine and aspartic acid 204 to leucine, boosting activity to 20.3% and 21.8% of the maximum activity at the respective temperatures. In general, as an exogenous enzyme preparation, RuXyn exhibits considerable potential for improving the ruminal fermentation and utilization of wheat straw in ruminants.

## Figures and Tables

**Table 1:** Effects of various regents on RuXyn’s activity (%).

Additives	Low Concentration	High Concentration
	1 mM	5 mM
Ca^2+^	109.21 ± 7.01	107.49 ± 7.97
Na^+^	103.34 ± 0.43	93.03 ± 3.47
Mg^2+^	77.36 ± 1.96	47.50 ± 2.91
K^+^	86.28 ± 5.82	93.15 ± 7.70
Zn^2+^	64.20 ± 0.82	21.98 ± 1.06
Cu^2+^	5.09 ± 0.12	3.20 ± 0.28
Mn^2+^	159.23 ± 9.97	173.20 ± 10.17
β-mercaptoethanol	85.30 ± 2.41	74.81 ± 5.53
Dithiothreitol	102.19 ± 0.44	119.67 ± 4.59
EDTA	57.21 ± 1.79	43.57 ± 4.26
SDS	74.54 ± 2.02	43.92 ± 5.08
	0.05% (*v*/*v*)	0.25% (*v*/*v*)
Tween-20	86.42 ± 4.90	73.72 ± 2.28
Triton X-100	73.13 ± 3.87	93.70 ± 3.38

The enzyme’s activity, in the absence of any additives, was considered to be 100%. Both 0.05% (*v*/*v*) and 0.25% (*v*/*v*) refer to the amount added as the percentage of the total reaction system volume. Each value represents the mean ± standard deviation of three replicates.

**Table 2:** Effects of RuXyn on ruminal digestion and fermentation parameters of wheat straw under in vitro incubation.

Item	Control	RuXyn	*p*-Value
IVDMD (%)	57.59 ± 1.61	66.34 ± 1.02	0.001
pH	6.53 ± 0.03	6.44 ± 0.01	0.007
Total VFA (mM)	73.13 ± 3.31	83.54 ± 3.17	0.017
Acetate (mM)	44.33 ± 0.51	46.75 ± 0.80	0.011
Propionate (mM)	13.19 ± 0.41	16.58 ± 0.34	<0.001
Butyrate (mM)	7.74 ± 2.67	7.71 ± 0.16	0.987
Isobutyrate (mM)	0.56 ± 0.51	0.51 ± 0.37	0.891
Valerate (mM)	1.66 ± 0.36	5.52 ± 1.96	0.029
Isovalerate (mM)	5.66 ± 0.25	6.47 ± 1.18	0.307
NH_3_-N (mM)	0.28 ± 0.03	5.80 ± 0.13	<0.001
CH_4_ (mmol)	1.36 ± 0.07	1.39 ± 0.03	0.667
Total bacteria (log10 of 16S rDNA copy numbers/mL)	8.32 ± 0.13	8.64 ± 0.04	0.016

Each value represents the mean ± standard deviation of three replicates.

## Data Availability

The data presented in this study are available on request from the corresponding author. The data are not publicly available due to privacy and confidentiality agreements as well as other restrictions.

## Supplementary Materials

The following supporting information can be downloaded at: https://www.mdpi.com/article/10.3390/ani14010118/s1, Figure S1: BLAST [protein] results of RuXyn based on the UniProt Knowledgebase database; Figure S2: Phylogenetic tree of RuXyn using neighbor-joining (NJ) method. Figure S3: Conserved domains analysis of RuXyn using conserved domain database of NCBI.

## Abbreviations

CDD, Conserved Domain Database; CDS, coding sequence; DS, Discovery Studio; DDGSs, distillers dried grains with solubles; EDTA, ethylenediaminetetraacetic acid; GH30, glycosyl hydrolase 30 family; HPLC, high-performance liquid chromatography; IPTG, isopropyl-β-D-thiogalactoside; IVDMD, in vitro dry matter digestibility; LSD, least significant difference; PBS, phosphate-buffered saline; PCR, polymerase chain reaction; RuXyn, xylanase from the rumen metagenome; SDS, sodium dodecyl-sulfate; SDS-PAGE, sodium dodecyl-sulfate polyacrylamide gel electrophoresis; VFA, volatile fatty acid; XOSs, xylooligosaccharides.
