# Supplementary material for: A Glycosyl Hydrolase 30 Family Xylanase from the Rumen Metagenome and Its Effects on In Vitro Ruminal Fermentation of Wheat Straw
Source: Animals (Basel). 2023 Dec 28;14(1):118. doi: 10.3390/ani14010118 (PMC10778502; doi:10.3390/ani14010118)
Supplement: Supplementary file 1 [file animals-14-00118-s001.zip › animals-2733319-supplementary.pdf]

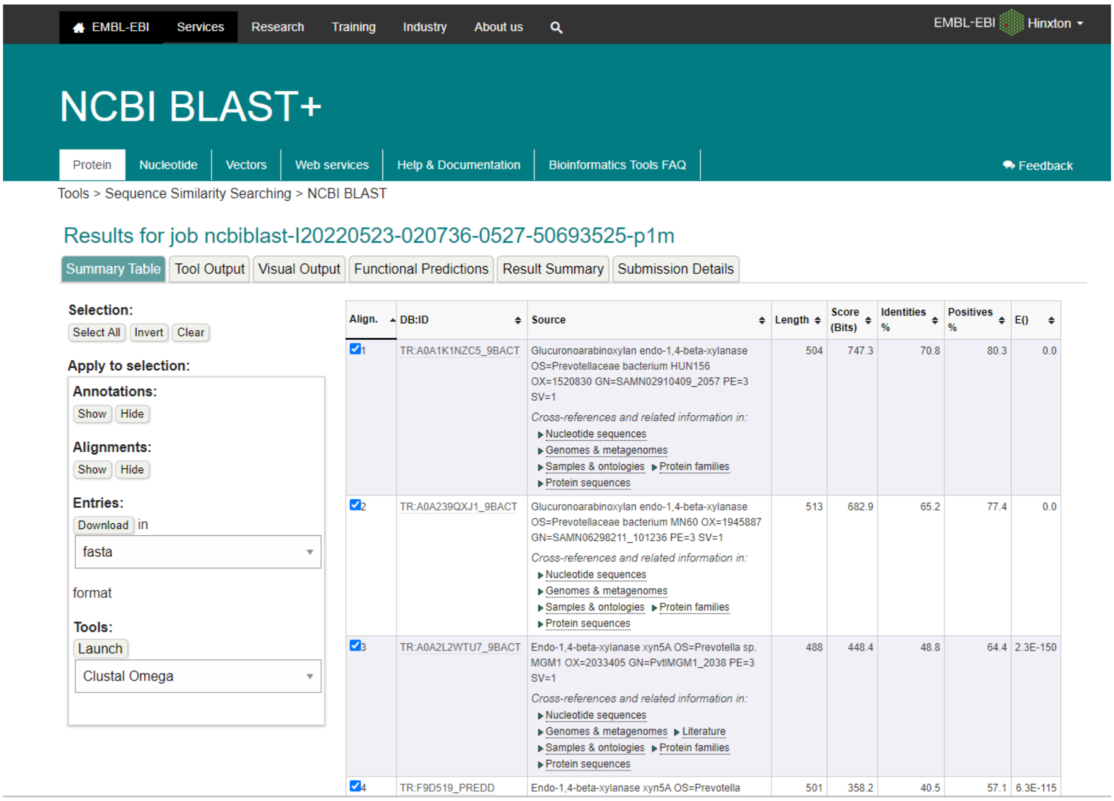

Figure S1. BLAST [protein] results of RuXyn based on the UniProt Knowledgebase database.

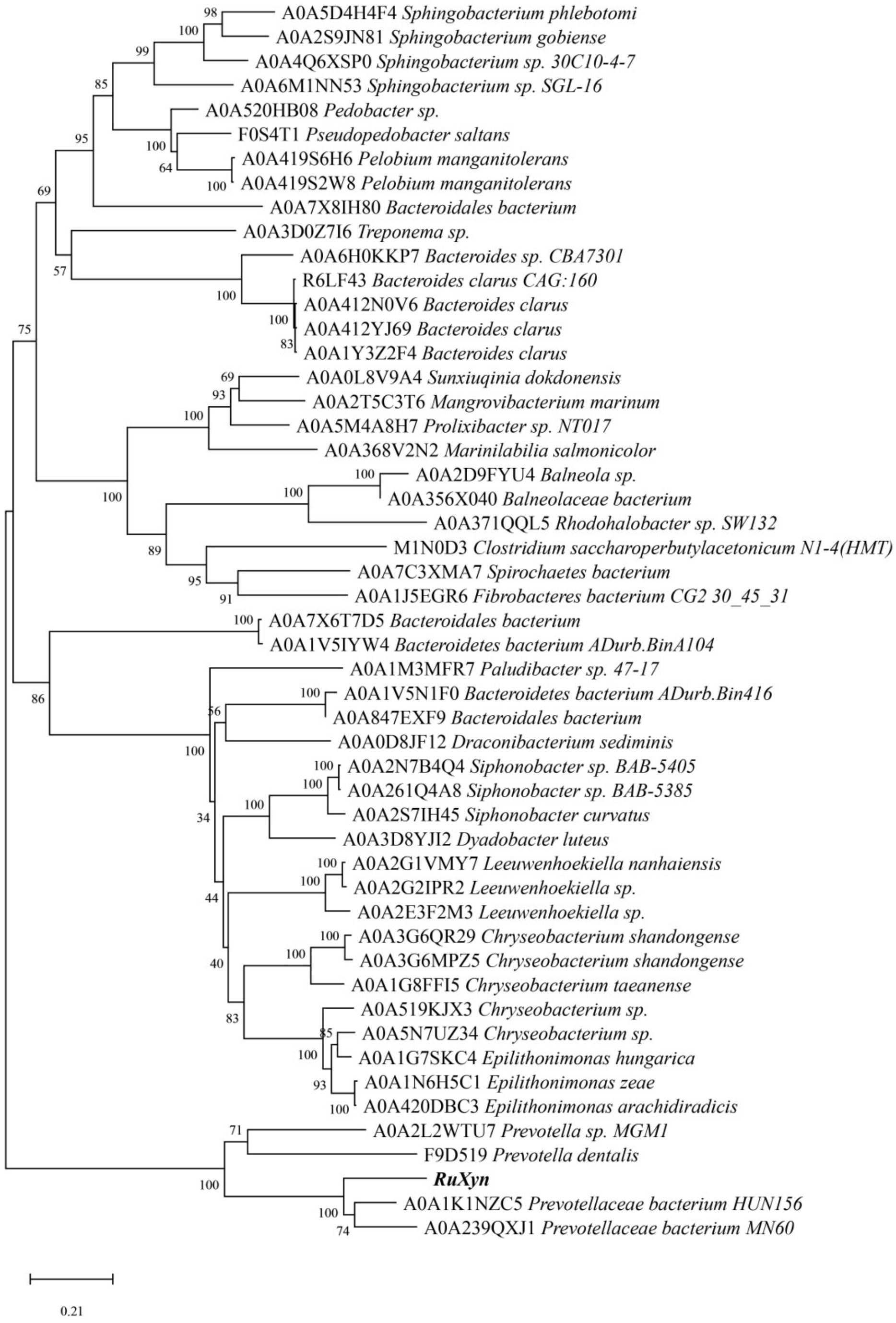

Figure S2. Phylogenetic tree of RuXyn using neighbor-joining (NJ) method.

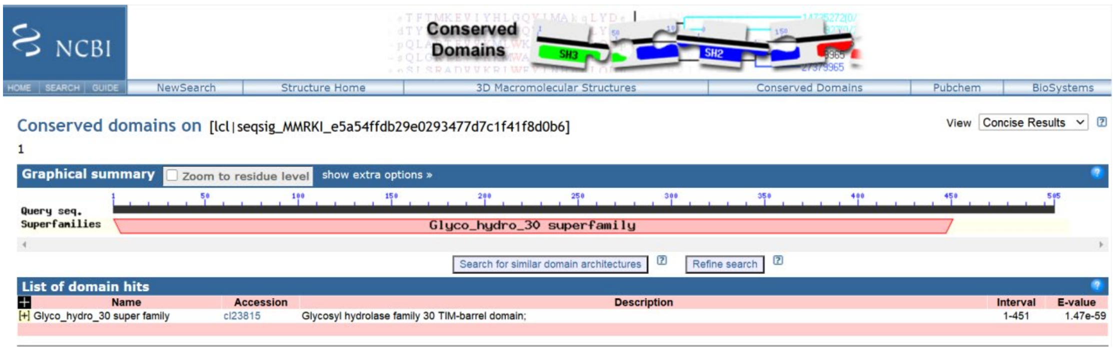

Figure S3. Conserved domains analysis of RuXyn using conserved domain database of NCBI.
